# Outcome of tibialis anterior tendon transfer in recurrent relapsed clubfoot deformity with concomitant peroneal nerve dysfunction

**DOI:** 10.12669/pjms.42.2.12956

**Published:** 2026-02

**Authors:** Anisuddin Bhatti, Syed Ata Ur Rahman, Saddam Mazar, Pervez Ali, Muhammad Yousuf Bhatti

**Affiliations:** 1 Anisuddin Bhatti, FCPS Orthopaedics. Consultant Orthopaedics & Paediatric Orthopaedic Surgery, Ziauddin University Hospital, Clifton Campus Karachi, Pakistan; 2 Syed Ata Ur Rahman, MS Orthopaedics. Fellow, Paediatric Orthopaedics, Ziauddin University Hospital, Clifton Campus Karachi, Pakistan; 3 Saddam Mazar, MS Orthopaedics. Ziauddin University Hospital, Clifton Campus Karachi, Pakistan; 4 Pervez Ali, FCPS Orthopaedics. Consultant Paediatric Orthopaedic Surgeon, NCCI Hospital / Jinnah Postgraduate Medical Centre, Karachi, Pakistan; 5 Muhammad Yousuf Bhatti, MBBS. Registrar, NCCI Hospital / Jinnah Postgraduate Medical Centre, Karachi, Pakistan

**Keywords:** Clubfoot, Iatrogenic complex clubfoot, Peroneal muscle dysfunction, Peroneal Nerve Dysfunction. Tibialis Anterior transfer

## Abstract

**Objectives:**

To assess the clinical and functional outcome of Tibialis Anterior Tendon Transfer in Recurrent Relapsed Idiopathic Clubfoot Deformity associated with peroneal nerve dysfunction.

**Methodology:**

This observational study included 16 (2.11%) of 756 Idiopathic Clubfoot Deformity (CFD) patients with recurrent and relapse deformity following Ponseti management, treated during January 1, 2011 to December 31, 2023 at Ziauddin Hospital Clifton, Karachi, Pakistan. Included patients had evertors and toe extensors weakness associated with concomitant Peroneal Neuropathy. Following an initial Ponseti re-casting, Extended Posterior Release (EPR) and Tibialis Anterior Tendon Transfer (TATT) was performed. Clinical and functional outcomes were assessed on Nogueira and Songs satisfaction protocols.

**Results:**

All 16 patients had bilateral idiopathic Clubfoot deformity at initial treatment, who developed Iatrogenic Complex Clubfoot deformity with concomitant Peroneal nerve dysfunction, four of these had recurrent relapse on left side only. Their muscle charting revealed peroneal and toe extensor muscles weakness, while Tibialis Anterior strength was grade IV. Five patients had flexible deformity, while 11 patients had fixed severe deformities. Thirteen patients underwent EPR and TATT, while three had Posterio-Medial Release and TATT. At 3-10 years of follow-up, six patients improved from Nogueira Poor to good Nogueira outcome and Satisfied on Song’s Scale. While three patients achieved Nogueira’s “Poor” and “Very dissatisfied” on Song’s criteria.

**Conclusion:**

A small and rare sub-set of iatrogenic complex clubfoot deformity with peroneal nerve dysfunction, is undoubtedly difficult to treat. Early diagnosis and treatment prevents extensive surgical interventions, which is required mostly in late presenting, rigid deformities. The association between peroneal neuropathy and preserved tibialis anterior muscle strength in these patients remains poorly understood.

## INTRODUCTION

Recurrence of varying severity in idiopathic Congenital Clubfoot Deformity (iCFD) after Ponseti treatment is not uncommon. The successful outcome of Ponseti treatment mounts as high as to 90% for initial correction, that declines to 60% - 80% with a relapse rate of 19% - 40%, in due course of time.^1^-^3^ Richard et al.^4^ reports, 37% relapse may occur within two years and Laaveg et al.^5^ reports 47% relapse occur within four years of age, while tendency to relapse considerably reduce after 6-7 years of age. Such decline in relapse is attributed to a more coordinated increase in activities, stretching of muscles during day and growth of muscles occurring at the same pace as in bone, among children in these age groups.^6^ Most of these relapses are attributed to host factors (type and severity of clubfeet deformity) and factors related to provider’s expertise including; inefficient casting, incomplete tenotomy, inadequate compliance to Foot Abduction Brace (FAB) and unexpected development of complex clubfeet.^1^,^2^,^7^

In the majority of iCFD relapses, repeated Ponseti serial casting followed by another set of Per Cutaneous Achilles Tenotomy (PCAT) has been found very effective, while atypical and complex CFD relapses also respond with modified Ponseti technique, when treated at earliest stage.^1^,^2^,^8^ However, some complex clubfoot deformities of iatrogenic cause, frequently exhibit relapses or remain poorly corrected despite re-treatments, PCAT or Tendo Achilles Lengthening (TAL) and compliance to bracing protocols. These iatrogenic complex CFD clinically exhibit varying severity of weakness of active dorsiflexion of ankle, toe and foot evertors due to having concomitant Peroneal Nerve Dysfunction (PND).^9^-^12^ The PND with Peroneal Muscle Dysfunction (PMD) is observed several months late in the course of treatment.^9^,^12^ The exact cause of PND-PMD however has not yet been well established in the context and controversies do exist, on the grounds that; such PND may not be the result of simple pressure of prolong castings around fibular neck causing infant peroneal palsy, as most of the compressive peroneal nerve dysfunction resolved within several months with a good prognosis, while others report none, partial to complete recovery of PND with restoration of Tibialis anterior muscle function.^9^,^10^,^12^

The response to treatment in recurrent-relapsed complex CFD, with concomitant PND, has been a challenging task.^9^,^11^ The early identification leads to better outcome with timely, more judicious, conservative approach, aimed to preserve the strength and range of motion at foot and ankle joints.^9^ Whereas, the late presenting fixed deformity cases, often require an extensive surgical procedures of TAL, ankle posterior capsulotomy and PMR with additional Tibialis Anterior Tendon Transfer (TATT) to restore active ankle dorsiflexion, that too with a compromised long term outcome.^9^,^11^ However in our cases, such patients received too late treatment during toddler’s age with a fixed biplane and triplane deformity.^1^ That include recommended procedures of articular extensive surgical intervention, TAL, additional bone wedge osteotomies and TATT, that significantly compromised the outcome to achieve a supple pain free feet at adulthood.^8^,^9^,^11^

The study is aimed to determine the fundamental cause of recurrent relapses in iCFD, developing Iatrogenic Complex CFD with concomitant PND, following a Ponseti treatment, re-treatment, and adherence to bracing protocol. Additionally, to evaluate the effectiveness of deformity correction surgery and TATT to 3rd Cuneiform. And to determine why majority of these cases reports late with fixed deformity, for surgical intervention, which was though advised at the earliest.

Study explains adequately with a literature review supporting that “Peroneal Nerve Dysfunction, occurs due to compression neuropathy of superficial and deep peroneal nerve (SPN-DPN) at fibular neck, often due to multiple attempts of Ponseti treatment for iatrogenic complex clubfoot deformity, the complete to partial recovery of such PND do occur in several months in course of the treatment.^11^,^13^ This do explain restoration of Tibialis anterior function and sensations, in our reported 16 cases. Whereas, the weakness of toe extensors and weakness of peroneal muscle function, could not be explained. In our hypothetical opinion “the weakness of toe extensors and peroneal function may be the result of muscle dystrophy of extensor digitorum brevis and peroneus tertius.” The dystrophy caused by repeated massage and manipulations as a part of Ponseti method. That is visibly evident as soft tissue atrophy at anterolateral aspect of the foot, just below lateral malleolus in majority of cases.

## METHODOLOGY

This observational study was conducted at Ziauddin Hospital Clifton (ZHC), Karachi, Pakistan. Given the retrospective nature, a consecutive sampling approach was used to ensure analysis of all relevant cases, whose data and photographs were available with corresponding author’s and his secured data maintained at International Clubfoot Registry (ICR-Pakistan). Patients were operated by corresponding author (ANB1) during January 1, 2011 to December 31, 2023 at ZHC and NCCI Hospital, Karachi.

### Ethical Approval:

It was obtained from the Ethical Review Committee (reference code 10040425ABORT; Dated: May 14, 2025).

Eligible criteria included patient having had typical, Idiopathic Congenital Clubfoot Deformity (iCFD), who developed iatrogenic complex CFD during course of initial Ponseti casting and bracing. Patients developed recurrent relapse or persisted with residual non-responsive CFD, despite undergoing 1-3 sessions of Ponseti castings and PCAT. The relapse deformity consistently exhibited equinus as a primary component, along with one or two other components of Cavus, Adductus, Varus, and Equinus (CAVE).^7^ Included patients having active weakness of the toe extensors and peroneal muscle. Following meticulous clinical examination, a neurodiagnostic studies with EMG-NCT was done and their reports revealed Peroneal muscle dysfunction (PMD) with a concomitant Peroneal nerve dysfunction (PND).^7^,^9^,^13^ To rule out possible additional lumbo-sacral nerve root pathology CT-MRI were also done. Patients were excluded if they had CFD associated with syndromic, paralytic, spastic, spinal dysraphism, myopathy and Sensory-Motor Polyneuropathy.^7^,^9^,^11^ Patients with unconfirmed PND/PMD, or a follow-up duration of less than one year were also excluded. Patients from study were also excluded if they did not come for in-person re-evaluation during last one year.

All patients initially underwent re-do Ponseti treatment to achieve at least plantigrade foot, with foot abduction of 15° or more. Thereafter patients in Bhaskar relapsed pattern IB-IIA with limited open TAL.^9^ The patients with Bhaskar IIb-III were operated with Extended Posterior Release (EPR) with or without posterior ankle joint capsulotomy. The EPR included TAL, lengthening of Tibialis Posterior, Flexor Digitorum and Flexor Hallucis tendons. Tibialis Anterior Tendon Transfer (TATT) to third Cuneiform was performed in all cases, to re-balance power and to provide dynamic internal splint. Whereas patients already had extensive TAL and in Bhaskar IIIB pattern Turco’s PMR was done.^9^,^11^

Patients with Residual Adductus and Cavus, an additional procedure of abductor Hallucis release, Steindler’s planter facial release and cuboid de-cancelation was carried out as an ala carte approach. Post-operatively immobilisation with an above knee cast was given for six weeks to secure TATT anchorage and correction achieved. The cast and anchorage button were removed at six weeks and a below knee walking cast applied for next 2-4 weeks. Gradual weight-bearing was allowed, followed by physiotherapy to restore strength of transferred Tibialis anterior muscle and to improve flexibility and suppleness.

Patients were evaluated with Nogueira et al “Outcome Classification”,^14^ and parents-patient satisfaction criteria defined by Song et al.^15^, at their last in-person follow-up. Clinical photographs and short video was recorded for re-evaluation by the two senior investigators; authors ANB^1^ & PA^4^. The extracted data from international clubfoot Registry (ICR) and corresponding author’s record, were systematically entered in a structured Case Report form (CRF) for each individual patient and thereafter in excel sheet for further evaluation as in Table-I-IV.

**Table-I:** Demographics of Patients and Pattern of Relapsed Clubfoot Deformity.

Case	Gender	Age			Laterally		Total Relapses	Bhaskar Pattern^1^	Nogueira Classification^14^
		1^st^ Cast (weeks)	1^st^ Relapse (Months)	Diagnosis of PMD (Years)	Initial Cast	Relapse			
1	Male	3	16	6.6	Bilateral	Left	5	II.B	Poor
2	Male	5	24	6	Bilateral	left	3	II.A	Regular
3	Male	1	15	7	Bilateral	left	4	III.B	Poor
4	Male	2	18	3	Bilateral	Bilateral	5	III.B	Poor
5	Male	1	18	10	Bilateral	Bilateral	2	III.B	Poor
6	Female	4	12	6	Bilateral	Bilateral	1	1.B	Regular
7	Male	2	12	7	Bilateral	Bilateral	3	III.A	Poor
8	Male	44	12	4	Bilateral	Bilateral	2	III.A	Poor
9	Male	46	24	3.6	Bilateral	Left	1	III.A	Poor
10	Male	4	12	5	Bilateral	Bilateral	2	II.B	Poor
11	Male	1	12	4	Bilateral	Bilateral	2	II.A	Regular
12	Female	2	12	5	Bilateral	Bilateral	2	III.A	Poor
13	Male	12	24	5	Bilateral	Bilateral	1	II.A	Regular
14	Male	24	60	10	Bilateral	Bilateral	1	II.A	Regular
15	Female	8	84	8	Bilateral	Bilateral	1	II.B	Poor
16	Female	2	60	5	Bilateral	Bilateral	2	II.B	Poor
PMD: Peroneal Muscle Dysfunction.									
Case #6,8,11,13 were treated elsewhere; #11recieved 18+10 cast + 1PCT; #13 Received 18+12 cast +1 PCT									
Mean Age at Initial Case			10.06+- 14.78 weeks, 96% CI [2.19, 17.93]						
Mean Age at 1^st^ Relapse			79.06 +- 89.02 weeks, 95% CI [31.53, 126.59]						
Nogueira Classification			Poor: 11(68.8%, 95% CI [0.461, 0.915]), Regular: 5 (31.2%, 95% CI [0.085, 0.539])						
Bhasker Pattern			II.B: 4 (25%, 95% CI [0.038, 0.462]), II.A: 4 (25%, 95% CI [0.038, 0.462]), III.A: 4 (25%, 95% CI [0.038, 0.462]), III.B: 3 (18.8%, 95% CI [0.000, 0.379]), I.B: 1 (6.2%, 95% CI [0.000, 0.182])						
Gender vs. Outcome			Fisher’s Exact Test, p = 1.000						
Relapses by Outcome			Mann-Whitney U Test, p = 0.15, Poor: Mean = 2.73, 95% CI [1.78, 3.68], Regular: Mean = 1.4, 95% CI [0.72, 2.08]						

**Table-II:** Distribution of Relapsed Clubfoot Deformities. Gender Versus Age Relationship and Laterality.

*Gender*	*Case No.*	Age				Relapse			Total Relapses	Bhaskar Pattern 1	Nogueira Classification 14
		1^st^ cast (Weeks)		1^st^ Relapse (Months)	Diagnosis of PMD (Years)	Bilateral	Left	Right			
Male	12	Min	1	12	3	8	4	0	Total 30 Avg 2.5	II.A (#2,11,13,14)	Regular: 4 (#2,11,13,14)
		Max	48	60	10						
		Total	145	247	71					II.B (#1,10) III.A (#7,8,9) III.B (#3,4,5)	Poor: 8 (#1,3,4,5,7,8,9,10)
		Avg	12	20.58	5.93						
		95% CI	[1.88, 22.28]	[13.73, 27.43]	[4.18, 7.66]				[1.76, 3.24]		
	
Female	4	Min	2	12	5	4	0	0	Total 6 Avg 1.5	I.B (#6)	Regular: 1 (#6)
		Max	8	84	8						
		Total	16	168	24					II.B (#15,16) III.A (#12)	Poor: 3 (# 12,15,16)
		Avg	4	42	6						
		95% CI	[1.67,6.33]	[7.44, 76.56]	[3.94, 8.06]				[0.81, 2.19]		

Total	16	Avg 10.06 Weeks Avg 2.51 Months		Avg 25.93 Months Avg 2.16 Years	Avg 5.9 Years Avg 5.9 Years	12	4	0	36 Avg 2.25 II.B: 4 III.A: 4 IIII.B: 3	I.B: 1 II.A: 4	Regular: 5
										Poor: 11	

Min: Minimum; Max: Maximum; Avg: Average; PMD: Peroneal Muscle dysfunction

Age at 1st Cast Mann-Whitney U Test, p = 0.03

Age at 1st Relapse Mann-Whitney U Test, p = 0.07

Diagnosis of PMD Mann-Whitney U Test, p = 0.95

**Table-III:** Outcome on Nogueira’s General Outcome Classification 14 and Song’s Parent-Patient’s Satisfaction.^15^

Nogueira’s Classification 14	Variable Criteria	Pre to Post-TATT Improvement			Song’s Satisfaction 15
		Pre-TATT	Post-TATT	95% CI (Post-TATT)	
Great (Excellent) (Bhaskar 0)	Foot fully plantigrade with at least 10 degrees of passive ankle dorsiflexion. Absence of any residual deformity or pain	0	0	[0.000, 0.000]	Very Satisfied
Good (Bhaskar 0)	Foot fully plantigrade. Dorsiflexion between 0 to 10 degrees. No residual deformity and free from pain	0	6 (37.5%) (#1,2,3,5,15,16).	[0.151, 0.599]	Satisfied
Regular (Bhaskar IA, B, IIA)	Little residual deformity and free from pain. Require additional surgery other than tibialis anterior transfer, or posteomedial release.	5 (31.2%) (#2,6,11,13,14)	7 (43.75%) (#6,8,9,11, 12,13,14)	[0.197, 0.678]	Ordinary (Equivocal)
Poor (Bhaskar IIB, IIIAB)	Failure of initial correction and need for extensive soft tissue release (posteromedial release)	11 (68.8%) (#1,3,4,5,7,8,9,10,12,15,16)	3 (18.75%) (#4,7,10)	[0.000, 0.387]	Very Dis-Satisfied

Wilcoxon Signed-Rank Test, p = 0.01.

**Table-IV:** Detailed Categories of Relapsed Deformities, Neuromuscular Status, Remedy and Outcome.

Bhaskar’s Pattern of Relapse 1	Relapsed CAVE Component	Case #	Previous Surgery	Remedy	Nogueira’s Outcome 14	Song’s Satisfaction 15
IB. Flexible deformity, Dynamic Supination.	Adductus, Varus	6	1PCAT	TATT, lateral wedge	Regular	Equivocal
IIA. Moderate deformity, fixed equinus	Varus, Equinus, Supination	2 (left), 11, 13, 14	2PCAT (left), 1 PCAT	EPR-TAL, TATT	Good (#2), Regular (#11, 13, 14)	Satisfactory (#2), Equivocal (#11, 13, 14)
IIB. Moderate Deformity, fixed equinus, varus or adductus. Fixed lateral curvature	Left, Adductus, Varus, Equinus	1 (left), 10, 15, 16	2PCAT, 1TAL (#1, #16), 1 PCAT 1TAL (#10), 1PCAT (#15)	EPR-TAL, TATT	Good (#1, 15, 16) Poor (#10)	Satisfactory (#1, 15, 16) Very Dissatisfied (#10)
IIIA. Severe Deformity, fixed Cavus Varus Equinus,	Cavus, Varus, Equinus	7, 8 (supination), 9 (left), 12	2PCAT, 1TAL (#7, 8) 1PCAT (#9) 2PCAT (#12)	PMR, SPR, TATT (#7); EPR-TAL, TATT, Lateral Wedge (#8); EPR-TAL, TATT (#9, 12)	Poor (#7); Regular (#8, 9, 12)	Very Dissatisfied (#7); Equivocal (#8, 9, 12)
IIIB. Severe Deformity, fixed Cavus, Adductus, Varus, Equinus,	Cavus, Adductus, Varus, Equinus	3 (left), 4 (Supination), 5	2PCAT,1TAL (#3); 2PCAT, 1TAL, 1TATT (#4); 1PCAT, 1TAL (#5)	EPR-TAL, TATT, SPR, Cuboid de-cancellation (#3); PMR, SPR, TATT (#4, 5)	Good (#3,5); Poor (#4)	Satisfactory (#3,5); Very Dissatisfied (#4)

Fisher’s Exact Test (Bhaskar Pattern vs. Outcome), p = 0.04, NEURO MUSCULAR STATUS: EMG-NCT revealed Peroneal Muscle dysfunction (PMD) and PND with Peroneal Nerve Axonal Neuropathy (PNANP) in all patients. MRI-CT was inconclusive in 14 cases and radiculopathy L4-5, L5-S1 in 2 cases (Ductal Ectasia (#4) and Lumbar 4-5 root Agenesis (#8). PCAT: Per-Cutaneous Achilles Tenotomy; EPR: Extended Posterior Release, TAL: Tendo Achilles lengthening, TATT: Tibialis Anterior Tendon Transfer. PMR: Posteromedial Release; SPR: Steindler Planter Release.

## RESULTS

Seventy (9.23%) cases out of our registered total 756 iCFD patients in ICR database of corresponding author (ANB1), had iatrogenic complex clubfeet, while 21 (2.77%) patients had recurrent relapsed CFD, with apparently weak ankle dorsiflexion, toe extensor and peroneal muscle weakness. EMG-NCT in these cases confirmed PNAN-PND. Of these 21 patients, five were dropped by not participated in prospective review, while 16 (2.11%) (28 feet) completed the latest follow-up. Further imaging with MRI-CT scan of spine revealed L4, L5 and S1 nerve root Pathology in two patient (4feet) while scans in other 14 cases were inconclusive. None of these patients had sensory loss on their first and subsequent visits (Table-I-IV). The majority of the patients 12 (75%) were males. All of them had bilateral idiopathic Clubfoot deformity initially, treatment commenced at minimum age of 1 week and at an average age of 10 weeks (Table-I-II). All these patients received 8-10 serial Ponseti castings and 1-2 percutaneous Achilles tenotomies for recurrent relapse. Eight patients required open TAL and one had TATT as well. All these patients had fair to poor compliance to bracing protocol, with history of frequent slippage of shoes due to persistent equinus.

The data of all most all these cases revealed; development of Iatrogenic Complex Clubfoot deformity before their first relapse. Twelves of these developed bilateral feet complexity (Chubby swollen feet, cockup big toe and Plantaris).^7^ While four cases (case #1,2,3,9) had unilateral complexity with recurrent relapse on left side only (Table-I-III). Muscle charting in all 28 feet revealed weak active toe extensors, poor functioning peroneal muscles, with reasonably good functioning Tibialis anterior muscle. The findings of PND (PNANP) in all the patients were diagnosed at a minimum age of three years and an average age of 5.9 years, after initial treatment (Table-I, II and IV). However, none of them had PMD or poor active dorsiflexion at initial Ponseti casting, as per record.

The majority of the patients 68.75% (11), had Bhaskar et al.^1^ IIB and III (Severe and fixed deformity pattern), comparable to Nogueira et al.^14^ classification as “Poor”. While 31.25% (5) patients had Bhaskar IB & IIA (flexible Equinus or varus deformity pattern with or without dynamic supination), comparable to Nogueira’s classification as “Regular” (Table-I and IV). One of them (#5), had repeated sessions of Ponseti casting, PCAT and bilateral TAL after 3^rd^ relapse. Diagnosed with PNANP at age of 10 years, developed progressive increasing CAVE deformity, gradually leading to a significant disability and overweight; making him unable to walk without walker for the last five years. Another patient #1, with bilateral CFD and a strong family history, developed left-sided fixed VAE deformity (Bhaskar IIB) due to early iatrogenic complexity, despite undergoing TAL. The right foot showed good correction (Bhaskar 0) over five years of follow-up.

The clinical functional outcome at 3-10 years follow-up revealed improvement from Nogueira’s class “Poor” to “Good” and satisfactory outcome with Song’s criteria in 6 patients (9 feet). Five (#1,2,3,15,16) of these patients had EPR-TAL and TATT, while 1 (#5) of them had PMR. They had Plantigrade feet with some residual varus, pain free weight bearing feet and using normal regular shoes. The other seven patients with 13 feet (#6,8,9,11,12,13,14) showed moderate improvement from Nogueira Class “Poor” - “Regular” to “Regular” with equivocal (ordinary) satisfaction on Song’s criteria. Whereas remaining 3 patients with 6 feet (#4,7,10,) could not improve, they persistently remained in Nogueira’s “Poor” class and were “Very dissatisfied” on Song’s criteria. All these three including two patients (#4,7) having lumber nerve root radiculopathy, persisted with nearly same deformity, rigidity, small plantigrade feet and significant calf thinness (Table-III-IV). Generally, none of patients could achieve clinically excellent improvement with very satisfied psycho-social impact, while majority (10 patients) of them either remained confused with equivocal satisfaction or persisted unhappy due to moderate to no clinical nor functional improvement of peronei and toe extensors.

Data analysis was performed using descriptive and inferential statistics. Continuous variables were expressed as means ± standard deviations and 95% confidence intervals, while categorical variables were presented as frequencies and percentages. The normality of data distribution was assessed using the Shapiro-Wilk test. Group comparisons of continuous data employed the Mann-Whitney U test, and paired change over time was evaluated with the Wilcoxon signed-rank test. For categorical variables, associations were analysed using Fisher’s exact test due to the small sample size (n=16). Statistical significance was set at p < 0.05, with exact p-values reported where applicable. Primary outcomes included Nogueira’s outcome criteria and Song’s parent-patient’s satisfaction at final follow-up (mean 8.5 years). Secondary outcomes involved radiographic findings and complication rates. Effect sizes and 95% CIs were calculated to support clinical interpretation. Analyses were conducted in SPSS v23.

## DISCUSSION

The retrospective review of our 758 cases with idiopathic CFD treated during last 15 years, we could find 9.23% (70 patients) having had iatrogenic complex clubfeet, while only 2.11% (16 patients with 28 feet) had apparently weak ankle dorsiflexion, toe extensor, peroneal muscle weakness and intact sensations. PND-PMD with PNANP was duly confirmed with EMG-NCT^9^,^10^,^12^,^13^,^16^ (Table-IV). This significantly low ratio of 2.11% patients with concomitant PND indicates rarity of such pathology as has been reported in literature by only few investigators, with a range varying 0.7% to 1.21%.^9^-^13^,^15^,^17^ Moreover, the peroneal compression neuropathy at the neck of fibula has been reported in literature as a cause of PMD; in 59% (10/17) cases of Jones et al.^12^, four of Gorden et al.^10^, six of Song et al.^15^, nine of Edmonds et al.^17^ and one of Yilmaz et al.^13^ Most of these neuropathy (PND-PMD) has been identified within due course of early treatment in infancy.^9^,^12^,^13^

Furthermore the recovery in such cases was also reported with complete resolution of neuropathy during next few months and that too with good prognosis for CFD correction, with non-surgical treatment.^9^,^10^,^12^,^13^ Few other investigators, reports no recovery of PND with generally unsatisfactory outcome and required extensive surgery.^9^,^11^,^17^ The PMD-PND in our patients, was established too late after 3-10 years of initial cast applied during infancy, following a failed repeat sessions of Ponseti casting and 2-3 set of PCAT for early to late-”fixed deformities”^1^,^3^ (Table-IV). Most of these cases were diagnosed to have PND, not until late in course of treatment during maintenance phase of bracing. However, their PND was partially recovered with complete restoration of Tibialis anterior function and weak toe extensors, though both were supplied by DPN. Peroneal muscle also recovered partially with partial recovery of SPN. Moreover, two of our 16 cases revealed additional Lumbo-sacral radiculopathy identified on CT-MRI. Edmonds^17^ and two other labelled these cases with lumber radiculopathy as an “alternative cause of PMD.^7^,^10^,^17^ One of the common findings in our cases was, the frequent slip of brace shoes due to puffy feet and mild to moderate equinous that developed with iatrogenic complexity, reported by others investigators as well.^7^,^8^,^18^,^19^

This frequent slip of shoes leads to non-compliance of bracing protocols and parents are blamed for that, instead to find reason for non-compliance. Other common phenomena observed in our cases was, frequent delayed consults, creating longer gaps to get treated relapses at early stage, despite advise given by then. The socio-economic status, fear of surgical intervention, work hour loss and distant travel embargos were the other major factor of failure to get early relapse treatment.^19^ Eleven of 16 cases in this study had Bhaskar pattern IIB-III fixed deformity, while five others had relatively flexible Bhaskar 1B-IIA deformity. They all were having significant weakness of active toe extensors without big toe drop sign^17^,^20^, spared tibialis anterior with grade IV strength, weak active abduction-eversion and intact sensations (Table-IV). While none of their reports revealed neuro-deficit at initiation of treatment. The variation in relapsing pattern, persistent weakness of toe extensors and peronei with sparing of tibialis anterior muscle function in our too late presenting cases can be explained to some extent with literature support, that partial to complete recovery of compressive neuropathy of DPN-SPN do occur several months thereafter.^9^,^10^,^13^,^17^

Furthermore, the persistent active weakness of toe extensors and peronei may be due to extensor digitorum brevis and peroneus tertius muscle dystrophy produced by repeated Ponseti massage, manipulation and casting sessions. This has also been reported by a recent native Publication 21 This hypothesis can be supported with existing clinically significant finding of atrophic skin and underlying soft tissues along antero-lateral aspect of foot just below lateral malleolus in in almost all our cases. This can be supported with finding of Gupta et al.^11^ as well, that clubfeet with PND had prominence of 5th, 4th, and/or 3rd metatarsal heads, which became more prominent with progression of age”.

It was a formidable challenge to achieve best possible outcome of a cohort of toddlers age, having passive, biplane to triplane fixed deformities of Bhaskar IIA-IIIB pattern (Table-I-IV), with significant loss in range of movement and callosities.^1^,^11^,^22^ Following an initial correction with repeat Ponseti casting as per recommendations by multiple investigators.^15^,^17^,^23^ We adopted a less aggressive surgical ala carte approach in 13 patients having already had 1-2 times PCAT, extensive surgical PMR in two patients who already had open TAL and minimum aggressive TAL and lateral wedge resection in other one patient, depending on the severity (Table-IV) of their Bhaskar pattern of triplane and biplane deformity respectively.^1^,^14^,^22^ Instead PMR we used less aggressive approach of Extended Posterior Release (EPR), as PMR behave poorly with significant medial scaring and relapsing rate.^24^ The EPR included ala carte approach; with TAL, limited lengthening of both flexors and tibialis posterior Z-plasty and if required posterior ankle capsulotomy, planter fasciotomy. EPR was aimed to preserve ankle-mid-tarsal complex mobility, to preserve the strength and range of motion the foot ankle joints.^8^,^9^,^11^,^23^,^25^ After deformity correction Tibialis Anterior Tendon Transfer (TATT) to 3^rd^ cuneiform was made as per recommendations to restore active dorsiflexion, provide a dynamic internal splint and to eliminate need of an orthosis in this cohort of toddlers.^11^,^17^,^25^,^26^ TATT has been the most preferred procedure of choice, due to its synergetic effect, direct straight alignment of pull, with a better excursion than Tibialis posterior tendon transfer.^11^,^12^,^26^

Yoshioka et al.^9^ reported satisfactory functional outcome with modified Ponseti casting in their 10 cases, who developed PND several months after casting initiated at 02 to 47 months age. Two of them received TAL and 1 more had TATT, with resolution of PND in 03 of these 10 feet only. Whereas similar to reports of Gupta et al.^11^ and Edmonds et al.^17^, We could not generally achieve an excellent clinical and functional outcome. Our patients ultimately achieved plantigrade feet, similar to Nogueira class^14^ “Good” and “satisfactory” Patient-parents satisfaction in 37.5%% cases on Song’s criteria.^15^ While 62.5%% of cases achieved Nogueira class “Regular” and “Poor” outcome with an “equivocal” (ordinary) to “Poor” satisfaction of parent-patients on Song’s criteria.

### Strength of the study:

Prospective evaluation of retrospective data, with a latest physical evaluation of patients made this study more reliable intra-observer study. The study opens a window for further research on the subject major; to establish reasons of (a) PND / PMD in non-responders, and recurrent relapsed in idiopathic Clubfoot deformity, (b) preservation of tibialis anterior with poor extensors of toes.

### Limitations:

The limitations include its retrospective single-surgeon design, moderate sample size, lack of a control group, intra-observer assessment reliability, and potential selection bias due to the majority of cases being referral cases, with potential impact of late-detected issues.

## CONCLUSION

A small and rare sub-set of iatrogenic complex clubfoot deformity with peroneal nerve dysfunction, is undoubtedly difficult to evaluate and manage with expected the best outcome. The late presenting fixed deformity relapse, often require an extensive surgical procedures of TAL, posterior capsulotomy of ankle or PMR and an additional TATT transfer to restore active ankle dorsiflexion. The extensive surgery PMR however leads to more scaring and ultimate stiff feet. This study reproduced EPR al a carte approach following few sessions of casting, along with TATT, as being significantly better approach then extensive PMR, in terms to preserve foot and ankle mobility and minimise another relapse, and to achieve parents satisfaction.

### Author’s Contribution

**AB:** Operating surgeon, Manuscript design, data evaluation for outcome, Manuscript final rewriting, submission and responsible for the integrity and accuracy of the study.

**AuR:** Data collection, data compiling, tabulation, first manuscript writing.

**SM:** Data re-compiling, statistical evaluation, tabulation, manuscript re-formation.

**PA:** Operating surgeon, Manuscript design, patients evaluation, data compiling, manuscript reviewing.

**MYB:** Data collection, patients re-evaluation, reference collection and citation re-reviewing.
